# Does natural and hybrid immunity obviate the need for frequent vaccine boosters against SARS‐CoV‐2 in the endemic phase?

**DOI:** 10.1111/eci.13906

**Published:** 2022-11-24

**Authors:** Stefan Pilz, John P. A. Ioannidis

**Affiliations:** ^1^ Department of Internal Medicine, Division of Endocrinology and Diabetology Medical University of Graz Graz Austria; ^2^ Departments of Medicine, Epidemiology and Population Health, Biomedical Data Science, and Statistics and Meta‐Research Innovation Center at Stanford (METRICS) Stanford University Stanford California USA

**Keywords:** booster, COVID‐19, epidemiology, risk, SARS‐CoV‐2, vaccine policy

## Abstract

The coronavirus disease 2019 (COVID‐19) pandemic has entered its endemic phase and we observe significantly declining infection fatality rates due to severe acute respiratory syndrome coronavirus 2 (SARS‐CoV‐2). On this background, it is crucial but challenging to define current and future vaccine policy in a population with a high immunity against SARS‐CoV‐2 conferred by previous infections and/or vaccinations. Vaccine policy must consider the magnitude of the risks conferred by new infection(s) with current and evolving SARS‐CoV‐2 variants, how these risks vary in different groups of individuals, how to balance these risks against the apparently small, but existent, risks of harms of vaccination, and the cost–benefit of different options. More evidence from randomized controlled trials and continuously accumulating national health data is required to inform shared decision‐making with people who consider vaccination options. Vaccine policy makers should cautiously weight what vaccination schedules are needed, and refrain from urging frequent vaccine boosters unless supported by sufficient evidence.

## 1 INTRODUCTION

As the coronavirus disease 2019 (COVID‐19) pandemic has moved into the endemic phase, current and future optimal vaccine policy is important to define.^1^ In late 2022, the use of a fourth vaccine dose has already become a contested issue.^2^ More generally, will booster doses be needed in future, and if so, at what time intervals, under what circumstances, and for whom? Answering these complex questions requires understanding the magnitude of the risks conferred by new infection(s) with current and evolving severe acute respiratory syndrome coronavirus 2 (SARS‐CoV‐2) variants, how these risks vary in different groups of individuals, how to balance these risks against the apparently small, but existent, risks of harms for different vaccines and vaccination schedules and how to fathom the cost–benefit of different options. The key issue underlying this decision‐making process is whether and how to consider natural immunity after SARS‐CoV‐2 infections and hybrid immunity derived from previous infections and vaccination. It is very likely that the large majority of the global population has been infected with SARS‐CoV‐2 at least once by late 2022.^1^ Excluding China, in other countries people who have never been infected have probably become a rarity. It is well established that previous SARS‐CoV‐2 infections induce a significant and long‐lasting protection against reinfections and even more so against severe COVID‐19.^3^, ^4^, ^5^, ^6^ Compared with vaccination by two doses, natural immunity was associated with a significantly higher protection against SARS‐CoV‐2 infections before the emergence of Omicron, when identical times have elapsed since the last immune conferring event.^3^, ^4^ For natural, hybrid and vaccine‐induced immunity, the protection against severe COVID‐19 is more sustainable than for SARS‐CoV‐2 infections per se.^4^ Would the accumulated immunity suffice or how often should it be strengthened? To help deliberate on this question, we dissect here issues of current risk from SARS‐CoV‐2 and of protection offered by natural infection and vaccines.

## 2 RISK FROM SARS‐COV‐2 INFECTION IN 2022

Infection fatality risk has been very low in 2022, and the majority of Omicron infections seem to be asymptomatic.^7^ This may be due to lower inherent mortality risk with Omicron and its subvariants and/or a consequence of the protection offered by vaccination and/or prior infection. The same applies to the risk of severe disease and hospitalization.

In Denmark, there was little viral spread until late 2021 and then massive infections with Omicron ensued in a population that had been widely vaccinated. Omicron infection fatality rate (IFR) until mid‐March 2022 was estimated to be only 6.2 (95% confidence interval (CI): 5.1–7.5) per 100,000 infections among apparently healthy people 17–72 years old.^8^ Compared with previous infection waves in Denmark, there was a very significant decline in IFR (Table 1).^8^, ^9^

**TABLE 1:** Infection fatality rates due to SARS‐CoV‐2 in Denmark in the general population^8^, ^9^

Age (years)	Weeks 11–42, 2020	Weeks 43, 2020–weeks 6, 2021	January–April 2022
	30‐day infection fatality rate per 100,000 infections (95% confidence interval)		
17–35	≤ 13.2	≤4.0	1.6 (0.9–3.1)
36–50	≤ 30.0	7.02 (5.95–8.32)	4.1 (2.6–6.6)
51–60	74.1 (55.6–137)	50.3 (40.4–64.5)	7.6 (5.2–11.3)
61–72^a^	281 (158–1686)	156 (114–228)	15.1 (11.5–19.9)

^a^ 61–69 for the infection waves in 2020 and 2021.

In populations with substantial prior exposure to SARS‐CoV‐2, reinfections (the vast majority occurring in the Omicron waves) had less than a quarter of the hospitalization risk and one‐tenth the mortality risk compared with the primary infections, for example, in Vojvodina, Serbia, only 1% of reinfections required hospitalization and the case fatality for reinfections was only 0.15%.^10^ Accounting for nonascertained infections, this suggests that IFR for reinfections at the population level may be <0.05%, even in people who have not been vaccinated and may be even modestly lower when 2 or 3 doses of vaccine have also been given.

## 3 PROTECTION OFFERED FROM PRIOR INFECTION

National data from Portugal including a population aged 12 years and older with an 82% coverage of the third vaccine dose against SARS‐CoV‐2 were used to compare groups of previously uninfected individuals versus groups with one documented infection in terms of infection rates with SARS‐CoV‐2 during the Omicron BA.4/BA.5 wave between 1 June and 4 July 2022.^11^ Three to 5 months after a previous BA.1/BA.2 infection, the effectiveness of protection against BA.4/BA.5 was 75.3% (95% CI: 75.0%–75.6%). Accordingly, individuals with previous infections with the Wuhan‐Hu‐1, Alpha and Delta variants had respective protection efficacies of 51.6% (50.6–52.6), 54.8% (51.1–58.2) and 61.3% (60.3–62.2), respectively. These data suggest long‐term protection by previous infections, when considering that the Wuhan‐Hu‐1 variant wave occurred at least one and a half year before the BA.4/BA.5 wave. Data from Qatar suggest that protection of natural immunity against any SARS‐CoV‐2 infection wanes over time and diminishes within a few years, while protection against severe, critical or fatal COVID‐19 remains strong and sustained.^12^ In detail, the effectiveness of a pre‐Omicron infection against any Omicron reinfection was 38.1% (95% CI: 36.3%–39.8%) and against severe, critical or fatal Omicron infection 88.6% (95% CI: 70.9%–95.5%). Effectiveness of protection of a previous infection with any SARS‐CoV‐2 variant against severe, critical or fatal COVID‐19 due to any variant was 97.3% (95% CI: 94.9%–98.6%), with no evidence of waning protection after 14 months.^12^ Out of 7082 documented reinfections, nine progressed to severe and only one to fatal COVID‐19.

Characteristics of immunity against SARS‐CoV‐2 on a national level may also be of interest for offering some hints on the relative protection offered by prior infection versus vaccination. For example, South Africa has had a low vaccination uptake in 2021 but high prevalence of previously infected individuals when considering seroprevalence data.^13^ The Omicron wave was very mild in South Africa compared with previous infection waves in that country.^14^ Conversely, the opposite applied to Australia, a country with a high vaccination uptake but low natural or hybrid immunity before Omicron. Moving forward, nevertheless, the intense debate on whether vaccination or natural infection is more protective becomes a largely moot question, since almost the entire global population has been already infected and >70% of the global population has also been vaccinated at least with some vaccine dose(s). Major epidemiological features on SARS‐CoV‐2 are summarized in Table 2.

**TABLE 2:** Major epidemiological features on SARS‐CoV‐2

The vast majority of the global population has already been infected with SARS‐CoV‐2.
Previous infections with SARS‐CoV‐2 significantly protect against reinfections and even more so against severe COVID‐19.
Infection fatality rates have significantly declined over the course of the COVID‐19 pandemic.
For natural, hybrid and vaccine‐induced immunity, the protection against severe COVID‐19 is more sustainable than against SARS‐CoV‐2 infections per se.

## 4 PROTECTION OFFERED BY A FOURTH VACCINE DOSE

There are currently no data on vaccine efficacies for the fourth dose (referring to mRNA vaccines) with reference to the prevention of SARS‐CoV‐2 infections from large RCTs, so we have to rely on observational studies. These studies have been largely conducted in Israel, as national health authorities in this country recommended a fourth dose of the BioNTech‐Pfizer vaccine (BNT162b2) for all individuals aged 60 years and older at the beginning of 2022, along with recommendations for this booster in immunosuppressed individuals and those at high risk of exposure (e.g. healthcare workers).^15^, ^16^ From 10 January to 13 March, 97,499 individuals aged 60 years and older (mean age ± SD: 70.8 ± 8.0 years) without any previous SARS‐CoV‐2 infection but with a PCR test during this time were investigated to compare SARS‐CoV‐2 infection rates in those who just received the fourth vaccine dose (*n* = 27,876) and those who had only received three vaccine doses (*n* = 69,623).^15^ In analyses stratified by time since the last vaccination, relative vaccine effectiveness with reference to any SARS‐CoV‐2 infection peaked during the third week at 65.1% (95% CI: 63.0%–67.1%) and declined to 22.0% (4.9%–36.1%) by the end of week 10. For severe COVID‐19, the respective efficacies after 7–27 days, 28–48 days and 46–69 days were 77.5% (69.7%–83.2%), 72.8% (58.8%–82.1%) and 86.5% (63.4%–95%), respectively. Of note, throughout the 10‐week follow‐up, only 572 of the 97,499 study participants had severe COVID‐19 (admitted to hospital or died due to COVID‐19) and only 106 patients died. Therefore, the numbers needed to treat (NNT) to save one life or one hospitalization can be very large. Another investigation from Israel was performed in 29,611 healthcare workers (65% female; age: 44 ± 12 years) without any previous SARS‐CoV‐2 infection.^16^ SARS‐CoV‐2 infections during January 2022 were documented in 7% of the participants with four vaccine doses and in 20% with three vaccine doses, resulting in a protection efficacy of 65% (95% CI: 61%–68%). In both groups, there was no severe COVID‐19 infection or death. The number needed to treat is therefore infinite for these serious outcomes. One has to wonder whether simply decreasing detected cases offers a clinically meaningful benefit.

In contrast to these healthcare professionals with an extremely low risk of severe COVID‐19, the respective risk may be much higher in very old populations with frailty and comorbidities.^17^, ^18^ This must be considered for vaccine policy as a similar relative vaccine efficacy translates into a clinically significant absolute risk reduction in populations with a high underlying risk for severe outcomes, but may be almost negligible in populations with a very low risk.^16^, ^17^, ^18^ Consequently, four versus three vaccine doses in residents of long‐term care facilities were associated with meaningful reductions in severe COVID‐19 and mortality and could justify recommendations for the fourth vaccine dose in this setting.^17^, ^18^ In detail, matched groups of long‐term care residents from Sweden without any previous SARS‐CoV‐2 infection (*n* = 12,262 for each group) were followed up for 7–60 days from January to May 2022.^17^ During follow‐up, there were 573 deaths (79.8 deaths per 100,000 person days) in the three‐dose group, and 292 deaths (105.2 deaths per 100,000 person days) in the four‐dose group, respectively, translating into a relative vaccine efficacy of 39% (95% CI: 29%–48%). The absolute benefit translates to approximately NNT = 100 during 40 days (1 life saved during 40 days of follow‐up per 100 doses given). However, what is essential to know is also the durability of these benefits with longer follow‐up. If the same benefit against mortality could be sustained for a year, then NNT would be 11. However, such sustained effectiveness is speculative.

A critical and serious limitation of the major studies in the general population on the efficacy of the fourth vaccine dose against SARS‐CoV‐2 is the almost universal exclusion of individuals with previous infections.^15^, ^16^, ^17^ The rationale for this exclusion criterion is usually not outlined, but the selection of an infection‐naïve population seriously limits the generalizability of the study findings now that almost everyone has already been infected with SARS‐CoV‐2. One exception is a study in 61,344 long‐term care residents from Ontario between 30 December 2021 and 27 April 2022, using a test‐negative study design and reporting on overall 3181 infections, including 606 symptomatic infections and 101 with severe outcomes.^18^ That study reported on a relative vaccine efficacy of four versus three doses of 19% (95% CI: 12%–26%) against any SARS‐CoV‐2 infection and of 40% (95% CI: 24%–52%) against severe outcome.^18^ In the same study, 15.6% of the residents with a negative PCR test had a previous SARS‐CoV‐2 infection, but only 7.5% with a positive PCR test. This suggests a significant protective effect by previous infections that may probably be higher than conferred by an additional vaccine dose.

## 5 VACCINE RISKS AND EVOLUTION WITH ADDITIONAL DOSES

The lower the underlying risk for severe COVID‐19 outcomes, the more careful should be the risk to benefit analyses of the vaccines, in particular if some adverse vaccine effects such as myocarditis are relatively frequent in low‐risk populations as, for example, male adolescents and young adults.^2^, ^19^, ^20^ Myocarditis and pericarditis have been reported at a rate of 52–137 cases per 1 million vaccinated male adolescents and young men after the second dose with BNT126b2 and mRNA‐1273, and at least 10 deaths have been attributed to these complications.^2^ There seems to be a mild decrease in the incidence of myocarditis after a third mRNA vaccine dose, whereas sufficient evidence has still to be generated regarding safety issues for further doses.^19^

## 6 COST–BENEFIT CONSIDERATIONS

Healthcare costs of mass vaccination and the possibility that overwhelming attention to SARS‐CoV‐2 could reduce resources for and adherence to other preventive measures such as uptake of other vaccines or health examinations might also be considered. A major challenge with factoring cost properly in such analyses is that many countries and conglomerates thereof, for example, the European Union, have made ludicrous massive contracts with vaccine manufacturers, with the lion's share pertaining to Pfizer for its mRNA vaccine and boosters. From a deal with the European Union, covering up to 1.8 billion vaccine doses, Pfizer/BioNTech may have made profits of up to 29.5 billion US Dollars.^21^ Some arrangements may stipulate the purchase of an extraordinary number of doses, skyrocketing public cost far beyond the cost of 3 or 4 total doses. Depending on that cost, even if further boosters are found to have some clinical benefit exceeding the risk of harms, their cost‐effectiveness must be carefully studied. If cost per person is not low, cost–benefit may be unfavourable except for small subgroups of the population.

Previous cost‐effectiveness analyses on measures against SARS‐CoV‐2 were generally based on overestimated IFR and may thus have been too optimistic.^22^ For the majority of the population, SARS‐CoV‐2 IFRs currently are so low that even if mRNA vaccines were to have 100% effectiveness for death and no adverse effects, their cost–benefit could be questionable or unfavourable, unless vaccine cost is brought down to negligible levels. This means that a healthcare system could use these same financial resources to obtain cost‐effective interventions to save more lives from other diseases.

For illustrative purposes, we used the IFR from Denmark at the beginning of 2022 and calculated the NNTs and costs to prevent one COVID‐19 death according to various different efficacies and costs for an extra booster dose (see Table 3).^8^, ^22^, ^23^ The presented estimates assume that 100% of the population will be infected eventually. In general, costs and NNTs have to be multiplied by the inverse of the percentage of infected individuals, for example, by 4 (1/0.25) if 25% of the population get infected (with equal probability with and without the extra booster). Our estimates also do not account for time‐varying vaccine efficacies and the likely further decline of the IFR towards the end of 2022. However, we can also not exclude a future increase in IFR, for example, if more lethal new variants of SARS‐CoV‐2 emerge at some point. The estimates also do not consider other potential benefits of vaccines such as, for example, prevention of hospitalization and potential harms of vaccination.

**TABLE 3:** Number needed to treat (NNT) and cost‐effectiveness for prevention of COVID‐19 deaths by an extra vaccine booster against SARS‐CoV‐2 using infection fatality rates from Denmark from 2022 and hypothetical vaccine efficacies and vaccine costs (per dose) and assuming all people are eventually infected.

Age group (in years)	Relative vaccine efficacies for the prevention of COVID‐19 deaths						
	10%	30%	50%	80%	90%	95%	99%
Number needed to treat to prevent one COVID‐19 death							
17–35	625,000	208,333	125,000	78,125	69,444	65,789	63,131
36–50	243,902	81,301	48,780	30,488	27,100	25,674	24,637
51–60	131,579	43,860	26,316	16,447	14,620	13,850	13,291
61–72	66,225	22,075	13,245	8278	7358	6971	6689
Costs to prevent one COVID‐19 death assuming vaccine costs of 5 US Dollars							
17–35	3,125,000	1,041,665	625,000	390,625	347,220	328,945	315,655
36–50	1,219,510	406,505	243,900	152,440	135,500	128,370	123,185
51–60	657,895	219,300	131,580	82,235	73,100	69,250	66,455
61–72	331,125	110,375	66,225	41,390	36,790	34,855	33,445
Costs to prevent one COVID‐19 death assuming vaccine costs of 20 US Dollars							
17–35	12,500,000	4,166,660	2,500,000	1,562,500	1,388,880	1,315,780	1,262,620
36–50	4,878,040	1,626,020	975,600	609,760	542,000	513,480	492,740
51–60	2,631,580	877,200	526,320	328,940	292,400	277,000	265,820
61–72	1,324,500	441,500	264,900	165,560	147,160	139,420	133,780
Costs to prevent one COVID‐19 death assuming vaccine costs of 120 US Dollars							
17–35	75,000,000	24,999,960	15,000,000	9,375,000	8,333,280	7,894,680	7,575,720
36–50	29,268,240	9,756,120	5,853,600	3,658,560	3,252,000	3,080,880	2,956,440
51–60	15,789,480	5,263,200	3,157,920	1,973,640	1,754,400	1,662,000	1,594,920
61–72	7,947,000	2,649,000	1,589,400	993,360	882,960	836,520	802,680

Although we present only a rough estimate and not an accurate cost‐effectiveness analysis, such considerations are required for the discussion on the justification of any measures against COVID‐19.^24^, ^25^, ^26^, ^27^ Economic evaluations including determining cost‐effectiveness thresholds are also considered an ethical necessity since every public expenditure has unwanted side effects in terms of shortening expenditures and probably life expectancies related to other health problems.^25^, ^27^ Common cost‐effectiveness thresholds per quality‐adjusted life year (QALY) gained vary significantly across different countries but are approximately 50.000–100.000 US Dollars for high‐income countries—or the gross domestic product (GDP) per capita.^25^ As shown in Table 3, in general, the NNTs for vaccinations against SARS‐CoV‐2 are relatively high compared with other interventions such as drugs for heart failure with NNTs of 11–24 to prevent one death, or an NNT of 2 for thoracoabdominal aortic aneurysm repair.^28^, ^29^ The costs per death averted also tend to be extremely high, except in very old age groups, based on the current cost per dose (approximately $20) and even if the costs were to decrease to $5 per dose. If the cost increases to $120 per dose (as recently considered for future marketing by Pfizer), cost‐effectiveness may become unfavourable even for many very old people (Table 3).^23^

## 7 MOVING FORWARD: OBTAINING RANDOMIZED EVIDENCE FOR UNANSWERED QUESTIONS

Both COVID‐19 vaccines and the virus will probably evolve in the future. The evolution of the virus towards new variants is largely unpredictable. The inherent fatality risk of new variants and their immune evasion against immunity conferred by prior infection and/or vaccination will be important to monitor and consider, as they may markedly change the balance in favour or against specific vaccination schedules. Therefore, genomic, epidemiological and clinical impact surveillance for SARS‐CoV‐2 will remain important in the endemic phase, in a similar fashion as it has been for influenza. There is even more uncertainty about the future role of different vaccines. COVID‐19 vaccine development has an active research agenda. New vaccines may emerge, including vaccines that have better effectiveness at blocking infection and transmission, features where the currently widely used COVID‐19 vaccines perform poorly at.^2^

In the meanwhile, there is an urgent need to address several research questions that are required to inform current vaccine policy. Ideally, these questions should be addressed by large RCTs and access to continuously accumulating national health data: Does the general population with hybrid immunity benefit from additional vaccine booster doses? Does prevention of positive SARS‐CoV‐2 PCR tests by vaccination justify their use even if there is no meaningful effect on severe COVID‐19 or mortality in terms of absolute numbers of events averted? How should one balance COVID‐19 vaccine policy in the context of other vaccine policies or public health measures in view of limited resources and the interaction of each measure? What is the cost‐effectiveness of different strategies? Cost‐effectiveness needs to consider also the possibility of wasted vaccines that are paid pre‐emptively by governmental authorities, but are then not used in the shifting sand evolution of COVID‐19. Arrangements of authorities with vaccine manufacturers should not include pledging upfront payment for an unrealistic number of doses. Transparency, accountability and protection from conflicts of interest are totally essential in the interplay between manufacturers and government officers, scientific advisors and other stakeholders who shape perceptions, expectations and public opinion pressure about COVID‐19 vaccines. Many other important research questions need to be answered to inform vaccine policies that are currently based on limited evidence. The continued lack of rigorous, randomized evidence raises serious concerns.

The performance of timely, large‐scale RCTs for addressing the effectiveness and harms of booster strategies is clearly feasible and even indispensable. Contrary to the argument that large RCTs take very long to complete, during the pandemic large RCTs like RECOVERY and SOLIDARITY provided conclusive evidence on the effectiveness of several treatments within 3–6 months of being launched.^30^, ^31^ The same principles and methods can be applied to vaccines, offering 3–6 month outcomes. Then, long‐term follow‐up evidence can continue to accrue. Observational vaccine studies are useful, but they have many caveats and limitations and it would be precarious to trust them when treatment effects are probably of small magnitude, as in the case of fourth dose boosters and beyond.^32^

## 8 DECISION‐MAKING

While rigorous evidence remains unavailable, authorities struggle in their decision‐making resulting in heterogenous national recommendations regarding the fourth vaccine dose in the general population (see Table 4). Complexity of vaccine policy is high and is continuously changing. Extreme recommendations may continue to be proposed and endorsed. For example, in Austria, a general recommendation was made by the National Vaccination Committee for a fourth vaccine dose against SARS‐CoV‐2 for all individuals aged 12 years and older on 31 August 2022, which then was modified on 16 September 2022 to a recommendation for all above 12 years ‘who want to protect themselves’ and in particular for those above 60 years old.^33^ In this context, it should be emphasized children and young healthy adults who may have already acquired hybrid immunity are at extremely low risk for severe COVID‐19, as discussed above.^34^, ^35^ It is unclear (and probably unlikely) at present that the COVID‐19 risk reduction by additional vaccination boosters outweighs the overall adverse effects in populations with a very low baseline risk such as in children and healthy young adults. Even if it does, cost–benefit considerations would be unfavourable. Therefore, we strongly argue to refrain from recommendations for mass vaccination in, for example, children and healthy nonelderly adults with a fourth vaccine dose, unless such a policy becomes supported by sufficient evidence. In Denmark, vaccination against SARS‐CoV‐2 for healthy children below the age of 18 years has been generally stopped, even for the first and second injections.^36^

**TABLE 4:** National recommendations regarding the fourth vaccine dose against SARS‐CoV‐2 for persons in the general population according to selected countries and age as of fall 2022^33^, ^36^, ^38^, ^39^, ^40^, ^41^, ^42^

Country	Age in years						
	<12	12–18	19–50	51–60	61–70	71–80	>80
Austria	No	Yes, ‘those who want to protect themselves’			Yes	Yes	Yes
Germany	No	No	No	No	Yes	Yes	Yes
UK	No	No	No	Yes	Yes	Yes	Yes
Denmark	No	No	No	Yes	Yes	Yes	Yes
the Netherlands	No	No	No	No	Yes	Yes	Yes
USA	No	No	No	Yes	Yes	Yes	Yes
Australia	No	No	No	Yes	Yes	Yes	Yes

With a broader view, during the endemic phase of COVID‐19, we have to consider the possibility that mass vaccination with frequent boosters may no longer be necessary for the majority of the population but only for certain risk groups, for example, the elderly and in particular long‐term care residents. It is conceivable that SARS‐CoV‐2 infections may soon follow a similar pattern as the other endemic human coronaviruses, with a first, usually mild infection in childhood and thereafter frequent but also generally mild infections in adulthood.^37^ Vaccine policy makers should weigh cautiously what vaccination schedules are needed, if any, after the end for the COVID‐19 pandemic.^1^

## CONFLICTS OF INTEREST

The authors report no conflicts of interest.

## FUNDING INFORMATION

There was no specific funding for this work.

## AUTHOR CONTRIBUTIONS

All authors have substantially contributed to the design and writing of this manuscript.
